# It’s complicated: criteria for policy decisions for the clinical integration of genome-scale sequencing for reproductive decision making

**DOI:** 10.1002/mgg3.130

**Published:** 2015-04-06

**Authors:** Benjamin S Wilfond, Katrina AB Goddard

**Affiliations:** 1 Trueman Katz Center for Pediatric Bioethics, Seattle Children’s Research InstituteSeattle, Washington; 2 Department of Pediatrics, University of Washington School of MedicineSeattle, Washington; 3 Center for Health Research, Kaiser Permanente NorthwestPortland, Oregon

Genome-scale sequencing is likely to play an increasing role in clinical practice over time. Exploratory research is currently underway that will allow for the development of an evidence base about the benefits, harms, costs, and impacts of clinical sequencing in terms of its effects on patients, the public, and the health-care system. This evidence base will be instrumental in health policy decisions, and will inform us about the clinical contexts where it is appropriate to offer or recommend such testing, the approach to disclose results to patients and families, and the approach to deciding on the downstream services that will be “covered benefits” for individuals or family members by private and public funders of health care.

Evidence-based medicine has been appropriately embraced by the medical community as a better approach compared to extemporaneous policy based on provider, consumer, and market forces. Evidence-based medicine is important for both primary and intensive care and its role is signaled by the creation of “practice guidelines” to determine what is “medically indicated.” But clinical evidence alone is insufficient to develop policy, and “medically indicated” obscures the implicit ethical dimension of many health policy decisions. An alternative “evidentiary model” is also based on evidence, but incorporates value-based judgments about the importance of risks, benefits, and costs to various stakeholders (Wilfond and Nolan 1993). With this evidentiary model, the same empirical data could be used to either support a policy to introduce a new technology, or to not do so, based on other contextual factors that incorporate stakeholder perspectives.

Implementation of genome-scale clinical sequencing offers the potential to improve care in a wide range of settings from guiding oncology treatment decisions to diagnosing the cause of intellectual disabilities in a child (Calvo et al. 2012; Dixon-Salazar et al. 2012; Need et al. 2012; Biesecker and Green 2014). Research is ongoing to identify the added value of integrating this technology into clinical care and to describe the occurrence of potential adverse consequences related to harms and costs. However, the underlying value aspect that must be considered in guiding policy development for genome-scale clinical sequencing may be most palpable when this approach is applied to carrier detection. Along with newborn screening (Levy 2014), carrier testing is one of the oldest population-based applications of genetics and its normative implications have been long appreciated. Reproductive decisions are intensely personal and the use of this information for pregnancy termination makes policy decisions even more complicated.

Clinical experience with carrier testing emerged in the 1970s with programs for sickle cell anemia and Tay Sachs Disease (Wilfond and Thomson 2000). The opportunity for population-wide carrier testing for cystic fibrosis (CF) focused policy makers’ attention keenly in 1989. In spite of some urgent calls for population testing, a more deliberative approach to policy development was adopted. Clinical trials to assess the impact of CF carrier testing were conducted in a wide range of contexts. An NIH Consensus conference in 1997 concluded that it was acceptable to offer CF carrier testing in the general population (Mennuti et al. 1999). In 2001, the American College of Obstetrics and Gynecology developed professional guidelines supporting the offering of testing (Grody et al. 2001). In short order, many primary providers began offering CF carrier testing and this was typically covered by third party payers. Once CF carrier testing was implemented broadly, more experience was gained about less common variants and the challenges of making phenotypic predictions (Mickle and Cutting 2000; Strom et al. 2002).

The clinical trials for CF carrier testing underscored the value-based aspect of policy decisions. Uptake of carrier testing was higher when it was offered during pregnancy, offered by a clinician, and immediately available. However, these observations did not result in policy recommendations to explicitly adopt such an approach because of the recognition that carrier testing is not like other medical recommendations where higher uptake is necessarily better (Ioannou et al. 2014). People may not want carrier testing because they may not consider CF important to know about prenatally, just as people have different preferences to learn the gender of a fetus. Criteria such as “medical indications” alone are not sufficient or clearly useful in determining policy for reproductive information. While the information is obtained through medical means, the meaning of the information for the family is personal and subjective. There are many options available to carrier couples, including adoption, donor gametes, preimplantation genetic diagnosis, prenatal diagnosis and termination, continuing pregnancy regardless of outcome, and choosing a different mate. Further, there is high variability in the social acceptance of these options among the public.

Calling out the value-based nature of CF carrier testing is not intended to be critical of the policy decisions directing providers to offer testing and funders to cover testing. The policy decision for CF carrier testing appeared to be driven by public interest combined with data about relative safety, and an explicitly normative view that it is valuable to provide information that can offer reproductive options. Further, efforts were made to provide educational materials about CF that were balanced to reduce the likelihood that some people would agree to test because they did not appreciate that people with CF, while experiencing more medical issues than many children, have meaningful and flourishing lives as much as others.

Genome-scale sequence-based carrier testing could offer information about hundreds of conditions, including some conditions that are more serious and others that are less serious than CF. While many of the conditions are individually much less common than CF (which occurs about once in 4000 births), cumulatively, we expect to identify as many, if not more, couples to be at risk for an affected child with some condition. Empirical confirmation of this hypothesis will require experience in the routine offering beyond that obtained during clinical trials, just as rare adverse drug reactions are not appreciated until clinical usage is widespread. But it is possible to predict that the cumulative frequency of the hundreds to thousands of Mendelian recessive or x-linked conditions will yield an impact not too far from that of CF. For example, a recent analysis of over 23,000 patients from various obstetric, genetics, and infertility clinics who received an expanded panel for carrier testing with over 400 causal Mendelian variants found that 24% of individuals were carriers of at least one of these variants, and 127 (0.55%) carrier couples were identified (Lazarin et al. 2013). Given that there are over 1000 known Mendelian recessive or X-linked conditions, we can expect that genome sequencing will yield the identification of an even higher percentage of carriers and carrier couples.

Yet, incidence alone is not sufficient to justify genome-scale sequence-based carrier testing. Data about potential harms such as false positives and downstream medical costs will be needed, and some of these data can be obtained through clinical trials. It is important to appreciate that what is “experimental” about sequencing is not the technology per se, but the knowledge and experience to determine responsible and appropriate use in a widespread clinical context. Especially when offered in the preconception context, carrier screening can strengthen reproductive autonomy and informed decision making by maximizing reproductive options, and presenting information in a setting with less time pressure and emotional stress than prenatal testing (Modra et al. 2010). Recent professional guidance from the American College of Medical Genetics and Genomics (ACMG) for expanded carrier screening recommends that patients should be able to “opt out” of testing for conditions with mild phenotype, variable expressivity, or incomplete penetrance, and “opt in” for adult onset conditions (Grody et al. 2013). This guidance recognizes variability in patient perceptions of disability and the “burden” associated with conditions. Clinical trials for carrier testing also require explicit patient-oriented outcomes because of the inherently personal meaning of the information.

We are currently conducting such a controlled trial in a small population recruited through Kaiser Permanente Northwest in Portland, Oregon. One key aspect of this trial will be the opportunity to learn about the downstream costs within a comprehensive health-care delivery system, as well as how clinical providers respond to such information. In addition, we are also developing an approach that explicitly acknowledges the social and subjective dimension of carrier testing. We allow participants to choose results based on broad categorizations: life span limiting, serious, mild, adult onset, and unpredictable conditions. (Table1) We hope to learn if such categorizations are meaningful to both patients and providers. Our rationale for this approach has a strong ethical underpinning for respecting diversity in patients’ worldviews and values, and is also consistent with professional guidance from the ACMG (Grody et al. 2013). While many people may prefer to receive information about all results or no results, some may make discriminating choices. It will be important to learn how best to develop a categorization that is meaningful to patients. We have taken a deliberative approach to developing our taxonomy of conditions but we expect that we will learn from experience how to do it better. We also appreciate that while many parents may request to receive all available information in the preconception context, how or whether that information is used during pregnancy will be variable. Further, psychosocial outcomes are relevant to health, and there may be a benefit from receiving information to provide reassurance or set expectations about a future pregnancy, even if the information does not inform reproductive decision making directly.

**Table 1:** Taxonomy of autosomal recessive conditions with examples

Category	Description	Example conditions	Carrier frequency
Shortened life span	Most children do not live past early childhood, even with medical interventions	Spinal muscular atrophy	1 in 54
		Tay sachs	1 in 27^1^
Serious	Most children will have medical problems that require regular medical visits, daily medications, carefully monitored diets, or surgeries; or will have serious problems with learning, vision, hearing, or mobility. Children may have shortened life spans into early adulthood	Cystic fibrosis	1 in 32
		Phenylketonuria	1 in 60
		Bloom syndrome	1 in 134^1^
Mild	Most children will have medical problems that require occasional extra medical visits, occasional medications, a slightly modified diet, or surgery; or will have mild problems with learning, vision, hearing, or mobility	Achromotopsia	1 in 123
Adult Onset	Few have any symptoms as children, but medical, behavioral, vision, or hearing problems may begin as adults	Hemochromatosis	1 in 9
		Alpha 1 Antitrypsin	1 in 12
Unpredictable	It is difficult to predict the outcome for many children. Some children will have more serious versions but others will have more mild versions, or no problems at all	Fanconi anemia	1 in 181

1 Ashkenazi Jewish population.

The data for our trial will serve as the foundation for the evidentiary approach to policy development for reporting carrier status from clinical sequencing. Additional trials will be necessary, but one key outcome from this current study will be to advance knowledge on whether our approach is respectful of people’s personal goals for their families. It will be important that we learn how to present information in a balanced way that reasonably reflects the range of experiences that parents have raising children with medical conditions. Carrier testing will never meet the traditional criteria of “medically indicated.” Unlike other situations where “medically indicated” tests have widely accepted clinical value, such as using blood pressure measurement to guide effective approaches to reducing hypertension, carrier testing for reproductive planning will always be more complicated.

Our policy goal is socially complicated because we need to develop an approach that increases reproductive choices when the information is meaningful, and avoids unnecessarily complicating an already challenging time for families. We need to remain vigilant that we can meaningfully portray the range of conditions and the range of impacts on families. Reproductive decision making is value based, and we expect that not everyone will be interested in such testing. But offering a broader range of reproductive options appears to have important social value and is a reasonable use of our medical resources.

## References

[b1] Biesecker LG, Green RC (2014). Diagnostic clinical genome and exome sequencing. N. Engl. J. Med.

[b2] Calvo SE, Compton AG, Hershman SG, Lim SC, Lieber DS, Tucker EJ (2012). Sci. Transl. Med.

[b3] Dixon-Salazar TJ, Silhavy JL, Udpa N, Schroth J, Bielas S, Schaffer AE (2012). Sci. Transl. Med.

[b4] Grody WW, Cutting GR, Klinger KW, Richards CS, Watson MS, Desnick RJ (2001). Laboratory standards and guidelines for population-based cystic fibrosis carrier screening. Genet. Med.

[b5] Grody WW, Thompson BH, Gregg AR, Bean LH, Monaghan KG, Schneider A (2013). Genet. Med.

[b6] Ioannou L, McClaren BJ, Massie J, Lewis S, Metcalfe SA, Forrest L (2014). Genet. Med.

[b7] Lazarin GA, Haque IS, Nazareth S, Iori K, Patterson AS, Jacobson JL (2013). Genet. Med.

[b8] Levy HL (2014). Newborn screening: the genomic challenge. Mol. Genet. Genomic Med.

[b9] Mennuti MT, Thomson E, Press N (1999). Screening for cystic fibrosis carrier state. Obstet. Gynecol.

[b10] Mickle JE, Cutting GR (2000). Genotype-phenotype relationships in cystic fibrosis. Med. Clin. North Am.

[b11] Modra LJ, Massie RJ, Delatycki MB (2010). Ethical considerations in choosing a model for population-based cystic fibrosis carrier screening. Med. J. Aust.

[b12] Need AC, Shashi V, Hitomi Y, Schoch K, Shianna KV, McDonald MT (2012). J. Med. Genet.

[b13] Strom CM, Huang D, Buller A, Redman J, Crossley B, Anderson B (2002). Cystic fibrosis screening using the college panel: platform comparison and lessons learned from the first 20,000 samples. Genet. Med.

[b14] Wilfond BS, Nolan K (1993). National policy development for the clinical application of genetic diagnostic technologies. Lessons from cystic fibrosis. JAMA.

[b15] Wilfond B, Khoury M, Burke W, Thomson E, Thomson EJ (2000). Models of public health genetic policy development. Genetics and public health in the 21st century: using genetic information to improve health and prevent disease.

